# Sterol Regulatory Element-Binding Protein Sre1 Mediates the Development and Pathogenicity of the Grey Mould Fungus *Botrytis cinerea*

**DOI:** 10.3390/ijms26031365

**Published:** 2025-02-06

**Authors:** Ye Yuan, Shengnan Cao, Jiao Sun, Jie Hou, Mingzhe Zhang, Qingming Qin, Guihua Li

**Affiliations:** 1 College of Plant Sciences, Jilin University, Changchun 130062, China; jlyuanye@126.com (Y.Y.); caoshengnan@dlou.edu.cn (S.C.); sunjiao18@mails.jlu.edu.cn (J.S.); nihaojiehou@163.com (J.H.); mzzhang@jlu.edu.cn (M.Z.); qingming.qin@health.missouri.edu (Q.Q.); 2 College of Fisheries and Life Science, Dalian Ocean University, Dalian 116023, China; 3 Christopher S. Bond Life Sciences Center, Department of Molecular Microbiology and Immunology, School of Medicine, The University of Missouri, Columbia, MO 65211, USA

**Keywords:** *Botrytis cinerea*, development, pathogenesis, conidiation, sclerotium, hypoxia adaptation

## Abstract

The grey mould fungus *Botrytis cinerea* is a dangerous plant pathogen responsible for substantial agricultural losses worldwide. The pathogenic mechanisms still have many unclear aspects, and numerous new pathogenic genes remain to be identified. Here, we show that the sterol regulatory element-binding protein Sre1 plays an important role in the development and pathogenicity of *B. cinerea*. We identified a homologue of gene *SRE1* in the *B. cinerea* genome and utilized a reverse genetics approach to create the knockout mutant Δ*sre1*. Our results demonstrate that *SRE1* is essential for conidiation, as Δ*sre1* produced only 3% of the conidia compared to the wild-type strain. Conversely, Δ*sre1* exhibited increased sclerotium production, indicating a negative regulatory role of *SRE1* in sclerotium formation. Furthermore, ergosterol biosynthesis was significantly reduced in the Δ*sre1* mutant, correlating with increased sensitivity to low-oxygen conditions. Pathogenicity assays revealed that Δ*sre1* had significantly reduced virulence, although it maintained normal infection cushion formation and penetration capabilities. Additionally, *SRE1* was found to be crucial for hypoxia adaptation, as Δ*sre1* showed abnormal germination and reduced growth under low-oxygen conditions. These findings suggest that *SRE1* mediates the development and pathogenicity of *B. cinerea* by regulating lipid homeostasis and facilitating adaptation to host tissue environments.

## 1. Introduction

Grey mould is a common and severe fungal disease affecting plants worldwide. It can manifest on various parts of host plants, including leaves, stems, flowers, and fruits, and is often prevalent in cool, humid climates [1]. This disease is particularly problematic in the greenhouse for the production of horticultural crops. Under conditions of low temperature (around 20 °C) and high humidity (relative humidity above 90%), once the disease occurs, no host plant is spared; in severe cases, it can lead to reduced crop yields or even total crop loss [2].

The occurrence of grey mould is not limited to the growth stages of field crops; it can also arise during the harvesting, storage, and transportation of fruits and vegetables. Consequently, the economic losses attributed to grey mould globally can reach between USD 10 billion and USD 100 billion annually [3]. According to a worldwide survey among plant pathologist, grey mould was ranked as the second most important fungal disease of plants, following rice blast disease [4].

The primary pathogenic fungus responsible for grey mould is *Botrytis cinerea*, which has a wide host range, capable of infecting over 1400 plant species across 586 genera [5,6]. The severe impact of *B. cinerea* has resulted in significant economic losses worldwide. The completion of the whole-genome sequencing and the development of various molecular techniques have established *B. cinerea* as an important model organism in the study of molecular plant pathology [4,7].

*B. cinerea* is a typical necrotrophic plant pathogen. Under natural conditions, conidia serve as the main source of initial and secondary infections in host plants. Upon germination, conidia form a germ tube that penetrates the host through structures such as appressoria or infection cushions [8,9]. After invading host cells, *B. cinerea* secretes various pathogenic factors, including cutinases, cell wall-degrading enzymes (Pme1, Pg1, etc.), toxins (botrydial and botcinic acid), oxalic acid, sRNAs, metal chelating proteins (Ibp, etc.) and cell death-inducing proteins (Xyg1, Rae, etc.) [3,10,11,12,13,14], which can kill the host or suppress its defence responses. This allows the pathogen to exploit the host’s nutrient (such as host cell wall components) for its growth and reproduction, leading to the formation of extensive mycelia and conidia. At the later stages of infection, the mycelium of *B. cinerea* forms sclerotium, a melanized resting body, which enables it to overwinter. The environmental conditions required for the production of conidia and sclerotia are often quite different. For the model strain B05.10, light can induce conidia production, while incubation in the dark promotes sclerotia formation [5].

Lipid homeostasis in mammalian cells is controlled by a family of sterol regulatory element binding protein (SREBP) transcription factors [15]. Hughes et al. (2005) first characterized the SREBP transcription factor Sre1 as a crucial oxygen sensor in fission yeast *Schizosaccharomyces pombe* [16]. Sre1 regulates the expression of genes involved in sterol biosynthesis (e.g., *ERG25*, which encodes a key enzyme involved in ergosterol biosynthesis), as a response to low level of sterol and oxygen availability. This dual role highlights the importance of Sre1 in maintaining cellular homeostasis under varying environmental conditions.

Sre1 plays important roles in orchestrating the transcriptional response to anaerobic conditions [17]. The binding of Nro1 to the prolyl hydroxylase Ofd1 is oxygen-dependent, regulating the stability of Sre1. This mechanism ensures that Sre1 levels are adjusted according to oxygen availability, thereby fine-tuning the expression of genes involved in sterol metabolism and other anaerobic processes [18]. Ergosterol acts as a critical regulator of Sre1 processing [19]. Sre1 and Mga2 are coordinately regulated, which is essential for the adaptive response to hypoxia [20]. The unassembled ribosomal protein uS12/Rps23 undergoes prolyl dihydroxylation, which affects the stability and function of Sre1, thereby influencing the hypoxic response and overall cellular adaptation [21]. Sre1 is also involved in the regulation of carotenogenesis in the red yeast *Xanthophyllomyces dendrorhous* [22,23]. This suggests that Sre1 may have diverse functions beyond sterol regulation.

Sre1 has been implicated in the virulence of several pathogenic fungi. For instance, in *Cryptococcus neoformans*, Sre1 is essential for oxygen sensing and sterol homeostasis, which are critical for its virulence [24,25]. The regulation of Sre1 in response to hypoxia allows *C. neoformans* to adapt to the host environment, enhancing its pathogenic potential [26]. Additionally, studies on *Magnaporthe oryzae*, the rice blast fungus, have shown that Sre1 is involved in the hypoxic response, which is crucial for its invasive growth within host cells [27]. In the insect fungal pathogen *Beauveria bassiana*, BbSre1 has been shown to control oxidative stress response and lipid homeostasis, further emphasizing the importance of Sre1 in fungal pathogenicity [28]. Furthermore, the role of Sre1 in *Clonostachys rosea*, a biocontrol agent, highlights its involvement in fungicide tolerance (such as prothioconazole, an inhibitor of sterol biosynthesis targeting the C14-demethylase Erg11) and antagonism, suggesting that Sre1 may play a role in the ecological fitness of fungi [29].

Comparative studies of Sre1 across different fungal species have highlighted its evolutionary conservation, with the exception of the absence of Sre1 in Microsporidia and most Basidiomycota species [30]. While the core functions of Sre1 in regulating lipid metabolism are conserved, variations in its regulatory mechanisms and target genes have been observed, reflecting the adaptation of different fungal species to their specific ecological niches [30]. This diversity presents opportunities for further exploration of Sre1’s role in fungal biology and its potential applications in biotechnology and medicine. Whether Sre1 plays an important role in the pathogenicity of *B. cinerea* remains unclear. Here, we identified a homologue of *SRE1* in *B. cinerea*. Disruption of this gene suppressed *B. cinerea* conidiation and pathogenicity but promoted its sclerotium production. We found that the knockout mutant Δ*sre1* exhibited low level of ergosterol biosynthesis and increased sensitivity to low-oxygen conditions, suggesting that *SRE1* mediates development and pathogenesis of *B. cinerea*, likely via maintaining lipid homeostasis and regulation of its adaptation to the host tissue environments.

## 2. Results

### 2.1. Identification, Knockout, and Genetic Complementation of the B. cinerea Gene SRE1

We used the *S. pombe SRE1* protein sequence (NP_595694.1) as a reference and identified a homologous gene BCIN_01g05780 in the *B. cinerea* genome. The open reading frame of *B. cinerea SRE1* consists of 2848 nucleotides, containing two exonic regions, with the coding region comprising 2679 nucleotides that encode 892 amino acids.

**Figure 1:**
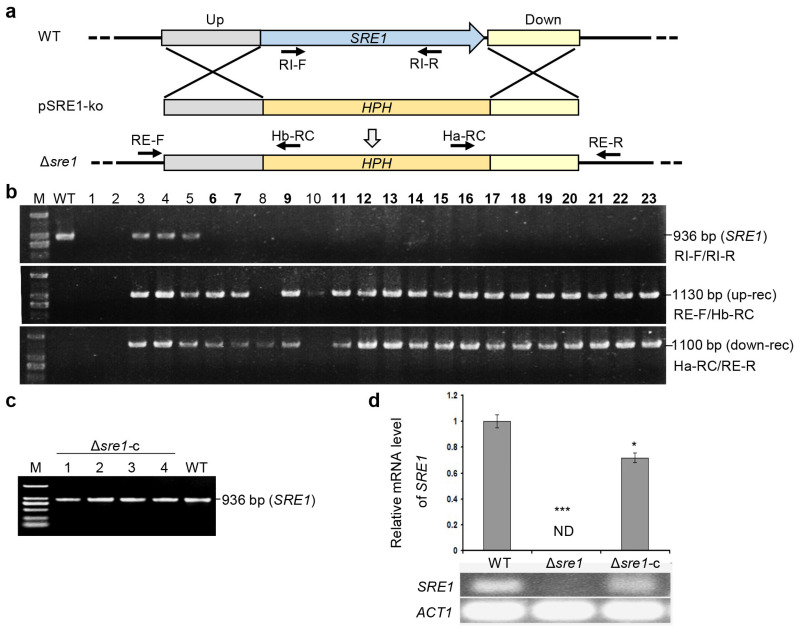
Generations of *Botrytis cinerea SRE1* knockout mutants and its genetic complemented strains. (**a**) Strategy for generation of *SRE1* knockout strain Δ*sre1* via gene replacement approach. WT, the wild-type strain B05.10; pSRE1-ko, *SRE1* knockout vector. *HPH*, the hygromycin resistance gene. (**b**) Screening of Δ*sre1* strains. Numbers 1–23 indicate partial selected transformants. PCR amplifications were used for detecting *HPH* recombination (rec) and *SRE1* loss in transformants, respectively, with indicated primers. Up-rec, upstream recombination; down-rec, downstream recombination. (**c**) Verification of the complemented strain Δ*sre1*-c. (**d**) Relative *SRE1* expression level in the indicated strains determined by quantitative reverse transcription PCR. M, DNA marker D2000. ND, not detected. Data represent means ± standard deviations (SD) from at least three independent experiments. *, ***, significance at *p* < 0.05, 0.001, respectively.

To confirm whether *SRE1* plays a crucial role in the growth, development, and pathogenicity of *B. cinerea*, we employed a reverse genetics approach to knock it out by replacement with the hygromycin resistance gene *HPH* (Figure 1a).

We introduced the knockout fragment into the wild-type strain B05.10 via the *Agrobacterium tumefaciens*-mediated transformation (ATMT) method. After screening for hygromycin resistance, we obtained a total of 105 transformants. PCR amplifications were used to identify knockout mutants. The transformants that tested positive for upstream and downstream recombination, and negative for the *SRE1* fragment were identified as the knockout mutant Δ*sre1*. Among these, 16 were confirmed as homozygous knockout mutants (Figure 1b), and mutant #21 was selected for further study.

To confirm the following observed defective phenotypes were indeed due to the deletion of *SRE1*, we constructed an ectopic genetic complemented strain, Δ*sre1*-c, based on the aforementioned Δ*sre1* mutant. We first amplified the complete *SRE1* gene (including its native promoter, coding region, and terminator) from the wild-type strain, then cloned and transformed it into the knockout mutant Δ*sre1*. After screening for G418 resistance, we obtained four transformants of Δ*sre1*-c (Figure 1c). Strain #1 was selected for further investigation. RT-PCR analysis indicated that *SRE1* mRNA was undetectable in Δ*sre1*, while its level in the complemented strain Δ*sre1*-c was recovered near to those of the wild-type strain (Figure 1d).

### 2.2. SRE1 Is Required for B. cinerea Conidiation but Dispensable for Conidial Morphogenesis and Germination

**Figure 2:**
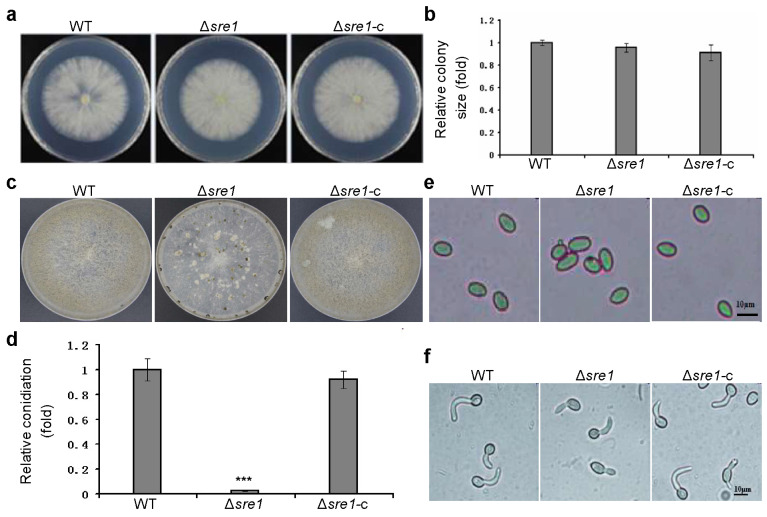
*SRE1* is required for *B. cinerea* conidiation but dispensable for conidial morphogenesis and germination. (**a**) Colony of tested strains cultured on PDA at 3 days post inoculation (DPI). (**b**) Quantification of the colony sizes (determined by the relative colony diameter). (**c**) Conidiation on CM plates at 12 DPI. (**d**) Quantification of the relative conidiation of the indicated strains (determined by the relative conidial number per plate). (**e**) Conidial morphology of tested strains. Bar = 10 μm. (**f**) Conidial germination of tested strains at 4 h post inoculation (HPI). Bar = 10 μm. Data represent means ± standard deviations (SD) from at least three independent experiments. ***, significance at *p* < 0.001.

To confirm whether *SRE1* is involved in the growth and development of *B. cinerea*, we first analyzed the growth of the Δ*sre1* on potato dextrose agar (PDA) plates. We found that both the colony morphology and growth rate were normal, showing no significant differences compared to the wild-type and complemented strains (Figure 2a,b). This indicates that *SRE1* is dispensable for vegetative growth.

Conidia are the primary means of dissemination and infection for *B. cinerea*. Next, we assessed the conidiation level of the mutant strain. Our results showed that after 12 days of cultivation on complete medium (CM) plates, Δ*sre1* produced significantly fewer conidia, amounting to only about 3% of the conidia produced by the wild-type B05.10 strain. In contrast, the complemented strain Δ*sre1*-c exhibited normal conidiation (Figure 2c,d). This result indicates that *SRE1* is required for *B. cinerea* conidiation. Additionally, we found that the absence of *SRE1* did not affect the morphology (Figure 2e) or germination (Figure 2f) of conidia.

### 2.3. SRE1 Mediates Sclerotium Production and Ergosterol Biosynthesis in B. cinerea

**Figure 3:**
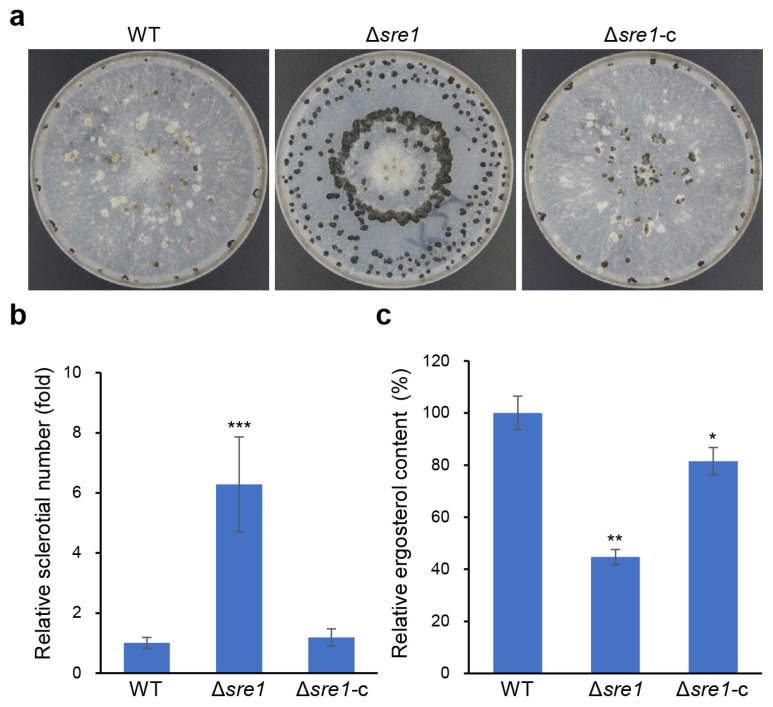
*SRE1* mediates sclerotium production and ergosterol biosynthesis in *B. cinerea*. (**a**) Deletion of *SRE1* in *B. cinerea* increases sclerotial production. The tested strains were cultured on CM at 20 °C in darkness and observed at 15 DPI. (**b**) Quantification of sclerotium production (determined by the relative sclerotial number per plate). (**c**) Quantification of ergosterol content (determined by the relative ergosterol content per gram of mycelium fresh weight). Data represent means ± standard deviations (SDs) from at least three independent experiments. *, **, ***, significance at *p* < 0.05, 0.01, 0.001, respectively.

Sclerotia play a crucial role in helping *B. cinerea* withstand adverse environmental conditions for completing its life cycle. To clarify the role of *SRE1* in sclerotium formation in *B. cinerea*, we inoculated wild-type B05.10, Δ*sre1*, and Δ*sre1*-c on CM plates and induced sclerotium formation by incubating them in the dark at 20 °C. The results indicated that the deletion of *SRE1* led to the production of a significantly higher number of sclerotia, approximately six times that of the wild-type and Δ*sre1*-c (Figure 3a,b), suggesting that *SRE1* negatively mediates sclerotium production.

In the fission yeast *S. pombe*, Sre1 acts as a transcription factor that promotes ergosterol biosynthesis when its level is low. To determine whether Sre1 in *B. cinerea* has a similar function, we measured the ergosterol content in the relevant strains. We found that the deletion of *SRE1* resulted in a significant reduction in the ergosterol content in mycelia, approximately half that of the wild-type strain. In contrast, the ergosterol level in the complemented strain was significantly restored (Figure 3c). This result indicates that Sre1 similarly regulates ergosterol biosynthesis in *B. cinerea*.

**Figure 4:**
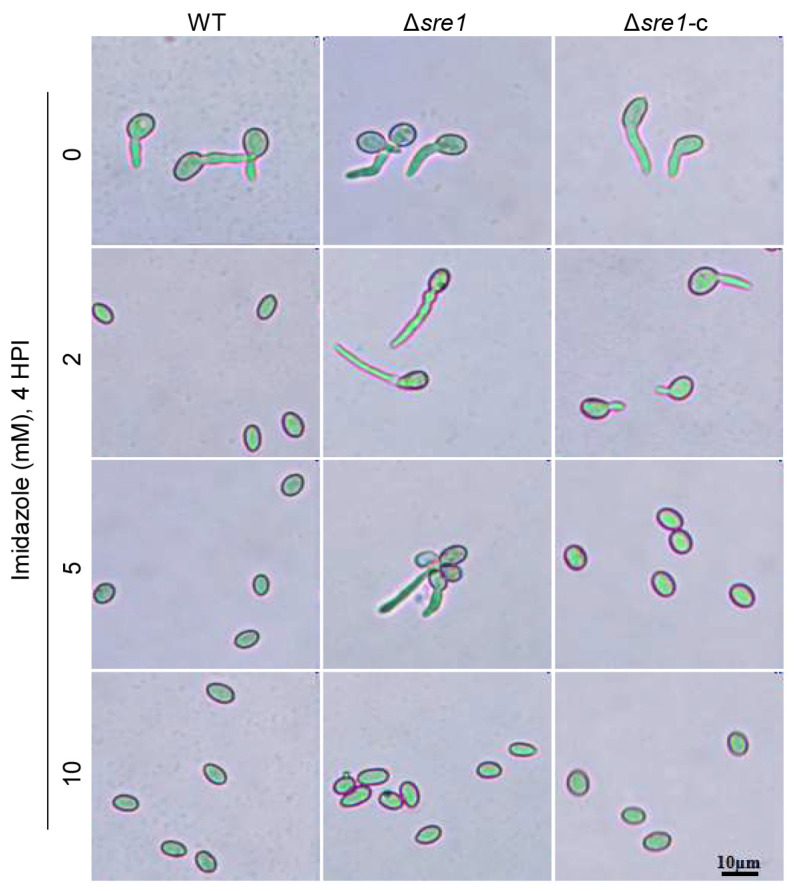
Deletion of *SRE1* increases *B. cinerea* resistance to antifungal drug imidazole. Conidial suspensions (containing 50 mM glucose) of each strain were treated with 0, 2, 5, and 10 mM imidazole, respectively. Conidial germinations were observed at 4 h post inoculation (HPI).

We treated the various strains with the antifungal drug imidazole to observe its effect on conidial germination. Surprisingly, the results showed that Δ*sre1* exhibited increased resistance levels (Figure 4; Figure S1), indicating that *SRE1* may negatively mediate resistance to this agent in *B. cinerea*.

### 2.4. SRE1 Is Required for B. cinerea Virulence but Dispensable for Its Infection Cushion Formation and Penetration

**Figure 5:**
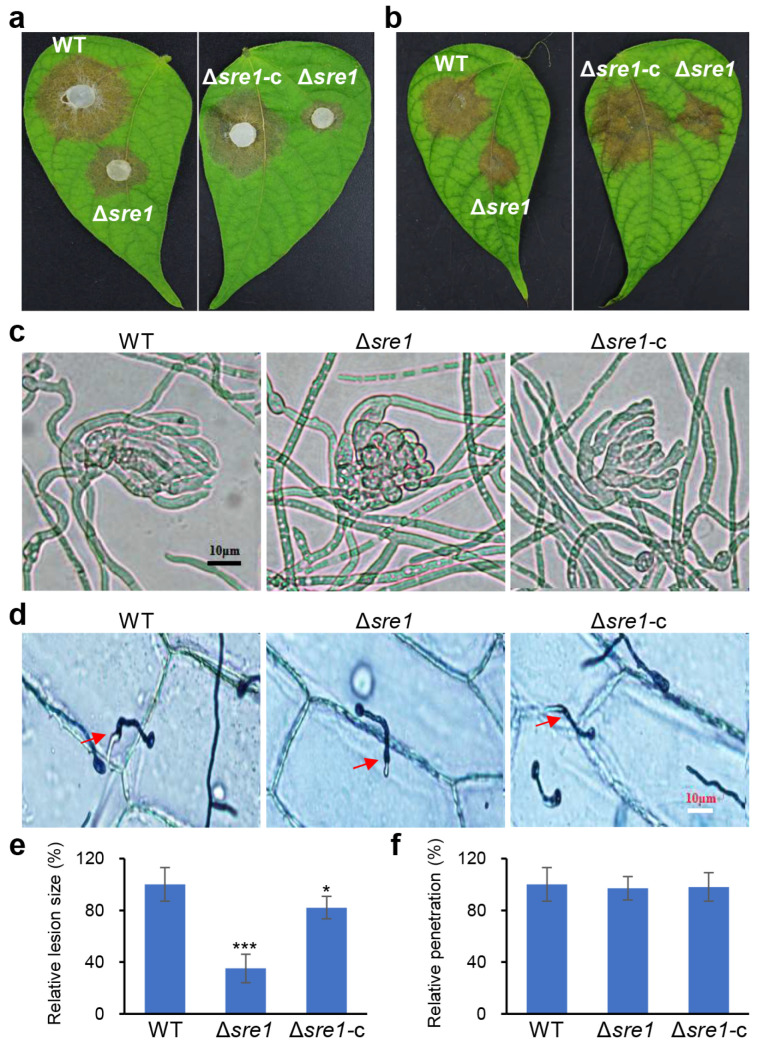
*SRE1* is required for *B. cinerea* virulence but dispensable for its infection cushion formation and penetration. (**a**) Mycelial plugs of each strain were inoculated on green bean leaves and the lesions were photographically documented at 3 DPI. (**b**) Conidial suspensions of each strain were inoculated on green bean leaves and the lesions were photographically documented at 3 DPI. (**c**) Infection cushion formation of tested strains at 20 HPI. (**d**) Onion epidermis penetration of the test strains at 12 HPI. Successful penetrations are indicated by red arrows. (**e**) Quantification of lesion size (determined by the relative lesion area per inoculation) caused by the indicated strains on green bean leaves shown in (**a**). (**f**) Quantification of penetration (determined per conidium) by the indicated strains on onion epidermis shown in (**d**). Data represent means ± standard deviations (SDs) from at least three independent experiments. *, ***, significance at *p* < 0.05, 0.001, respectively.

To verify whether *SRE1* is involved in the pathogenic process of *B. cinerea*, we inoculated detached green bean leaves with mycelial plugs from the wild-type strain B05.10, Δ*sre1*, and Δ*sre1*-c, respectively. At 72 h post inoculation (HPI), we observed that the lesion size caused by Δ*sre1* was significantly reduced compared to the wild-type B05.10, with only about one-third size of the wild-type lesions. In contrast, the pathogenicity of the complemented strain Δ*sre1*-c was significantly enhanced compared to the mutant (Figure 5a,e). Similar results were obtained using conidial suspensions for inoculation (Figure 5b). These results indicate that *SRE1* plays an important role in the pathogenic process of *B. cinerea*.

Infection cushion is a crucial structure for *B. cinerea* to penetrate the host. To confirm whether the reduced pathogenicity of Δ*sre1* is related to the development of the infection cushion, we inoculated conidial suspensions of the wild-type B05.10, Δ*sre1*, and Δ*sre1*-c onto glass slides to induce the formation of infection cushions. The results showed that Δ*sre1* could form infection cushions normally, and their morphology was also normal (Figure 5c). We further analyzed the penetration ability of the relevant strains on onion epidermis and found that the penetration capability of the mutant was also normal, with no significant differences compared to the wild-type and complemented strain (Figure 5d,f). These results indicate that the deletion of *SRE1* did not affect the early infection process of *B. cinerea* (including the development of infection structures and penetration), implying that *SRE1* may mediate *B. cinerea* pathogenicity by participating in the regulation of its adaptation to the host tissue environment after penetration.

### 2.5. SRE1 Is Involved in Hypoxia Adaptation of B. cinerea

**Figure 6:**
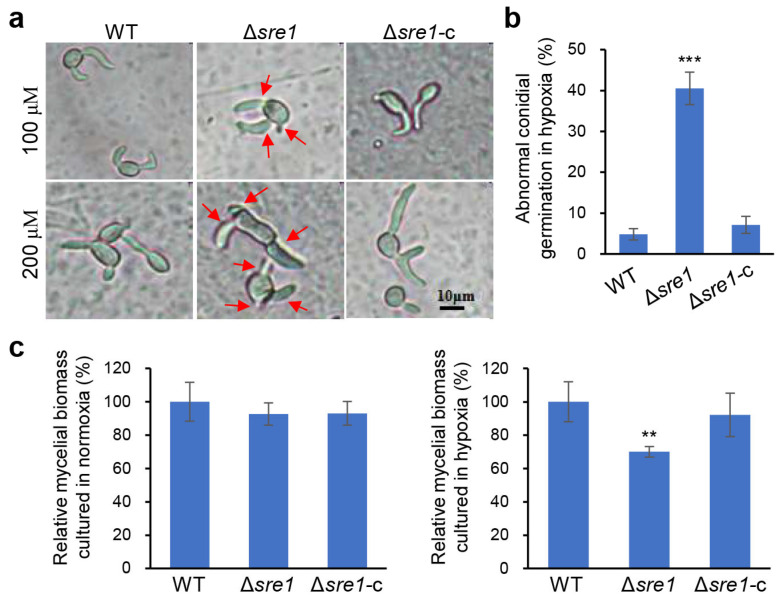
*SRE1* is involved in hypoxia adaptation. (**a**) Conidial suspensions (containing 50 mM glucose) of each strain were treated with 100 or 200 μM cobalt chloride (CoCl_2_), a hypoxia-mimicking agent. Conidial germinations were observed at 4 HPI. Germ tubes are indicated by red arrows. (**b**) Quantification of abnormal conidial germination in hypoxia-mimic with 100 μM CoCl_2_. (**c**) Quantification of mycelial biomass cultured in normoxia or in hypoxia-mimic with 100 μM CoCl_2_. Data represent means ± standard deviations (SD) from at least three independent experiments. **, ***, significance at *p* < 0.01, 0.001, respectively.

In the fission yeast *S. pombe*, the SREBP transcription factor Sre1 serves as a crucial oxygen sensor and plays important roles in orchestrating the transcriptional response to anaerobic conditions [17]. To determine whether Sre1 of *B. cinerea* is also involved in hypoxic response, we first treated the conidia of relevant strains with the hypoxia-mimicking agent cobalt chloride (CoCl_2_) to observe their germination under low-oxygen conditions. CoCl_2_ has been widely used as a hypoxia-mimicking agent in many organisms [31]. The results showed that the germination of Δ*sre1* conidia was abnormal, with a significantly higher proportion of conidia germinating with three germ tubes compared to the wild-type and complemented strains, which typically only with 1–2 germ tubes (Figure 6a,b).

Next, we treated the mycelia of each strain with the same hypoxia-mimicking conditions in liquid media. Quantitative analysis revealed that the growth of Δ*sre1* under low-oxygen conditions was significantly lower than that of the wild-type and complemented strains (Figure 6c, right panel). However, under normal conditions, there were no significant differences in growth among the strains (Figure 6c, left panel). These results indicate that *SRE1* plays an important role in the hypoxia adaptation of *B. cinerea*.

## 3. Discussion

In this study, we demonstrate that the sterol regulatory element-binding protein Sre1 is crucial for the development and pathogenicity of the grey mould fungus *B. cinerea*. Our findings provide new insights into how *SRE1* mediates key aspects of fungal biology, including conidiation, sclerotium formation, virulence, and adaptation to host environments. These processes are tightly connected to lipid homeostasis, particularly ergosterol biosynthesis, and the ability of *B. cinerea* to cope with hypoxic conditions during infection.

Our finding indicates the significant role of *B. cinerea SRE1* in regulating ergosterol biosynthesis. In the Δ*sre1* mutant, ergosterol content was reduced by approximately 50%, which aligns with the known function of *SRE1* in regulating sterol biosynthesis pathways in other fungi [16,32]. Ergosterol, the primary sterol in fungal membranes, is crucial for maintaining membrane integrity and fluidity, which is essential for various cellular processes including growth, signalling, and stress responses. Reduced ergosterol levels in the mutant strain Δ*sre1* may explain some of the observed developmental defects and altered stress responses, such as increased resistance to the antifungal drug imidazole, which targets ergosterol biosynthesis [32]. This observation could open new avenues for exploring how *B. cinerea* manages drug resistance and lipid metabolism during pathogenesis.

A striking observation from the study is that *SRE1* is essential for *B. cinerea* conidiation. The Δ*sre1* mutant exhibited severely reduced conidiation (only about 3% of the wild-type level), highlighting the importance of *SRE1* in fungal reproduction and dispersal. Conidiation is a critical step in the lifecycle of *B. cinerea*, as conidia serve as the primary infectious propagules, facilitating pathogen spread [4]. An earlier study has shown that the mutant of *M. oryzae SRE1* exhibits increased conidiation [27]. These finding indicate that *SRE1*s regulate conidial formation in a species-specific manner among fungi.

Sclerotium production is another key aspect of *B. cinerea* biology that is regulated by *SRE1*. This study revealed that Δ*sre1* mutant produces significantly more sclerotia, a structure crucial for the pathogen’s survival under unfavourable conditions [5]. This finding suggests that *SRE1* negatively regulates sclerotium formation, possibly through its role in sterol biosynthesis and lipid homeostasis. This suggests that Sre1 may act as a switch, promoting conidiation while inhibiting sclerotium formation, a strategy that helps the fungus adapt to changing environmental conditions. In contrast, lack of *SRE1* may shift the balance toward survival strategies, such as enhanced sclerotium formation, at the expense of reproductive dispersal (conidiation). The possibility that the enhanced sclerotia formation in the *SRE1* mutant is an indirect effect of reduced conidiation cannot yet be excluded, because the induction conditions for their development are quite different in the wild-type strain B05.10 [5], which suggests that their development may be antagonistic.

Our finding shows that *SRE1* plays a role in hypoxia adaptation of *B. cinerea*. In line with previous work in other fungal species, where *SRE1* is a key regulator of the response to low-oxygen conditions [16,17,26,27], we observed that the Δ*sre1* mutant exhibited impaired conidial germination and mycelial growth under hypoxic conditions. The use of CoCl_2_, a commonly used hypoxia-mimicking agent [21], revealed a distinct germination phenotype, with a higher proportion of conidia producing multiple germ tubes, which could be indicative of abnormal metabolic responses in the absence of *SRE1*. Additionally, the growth of the mutant in liquid culture under low-oxygen conditions was significantly reduced compared to the wild-type and complemented strains, further underscoring the critical role of *SRE1* in the hypoxic stress response. These results suggest that *SRE1* is a central player in the fungal adaptation to oxygen-limited environments during infection, a phenomenon that has also been highlighted in *C. neoformans* [26].

The role of *SRE1* in pathogenicity was another focal point of our study. The Δ*sre1* mutant exhibited significantly reduced virulence of *B. cinerea*. This reduced pathogenicity occurred despite normal formation of infection cushions and penetration ability, indicating that *SRE1* does not affect the initial stages of infection, but may instead be important for the pathogen’s adaptation to the host environment post-penetration. Similar observations were reported in *M. oryzae*, where the hypoxic response controlled by *SRE1* was found to be critical for its invasive growth within host cells [27]. Thus, our results suggest that *SRE1* mediates *B. cinerea* pathogenicity through its role in lipid homeostasis and adaptation to the hypoxic conditions typically encountered in host tissues.

The findings from this study open several interesting avenues for future research. First, further investigations into the molecular mechanisms by which *SRE1* regulates sclerotium formation and conidiation are needed. It would be valuable to explore whether *SRE1* influences these processes directly through gene regulation or indirectly through its impact on lipid metabolism. Additionally, the role of *SRE1* in fungal interactions with plant immune responses remains an important area for exploration. Studies could investigate whether and how *SRE1*-mediated lipid homeostasis affects the secretion of virulence factors and immune-modulating proteins, as seen in other pathogeneses [3]. Lastly, given the central role of *SRE1* in fungal hypoxic responses, it would be interesting to assess the potential of *SRE1* as a therapeutic target for controlling *B. cinerea* and other plant pathogens, especially considering its involvement in virulence and adaptation to stress conditions [28].

In conclusion, this study demonstrates that *SRE1* plays a multifaceted role in the development and pathogenicity of *B. cinerea*. It regulates key processes such as conidiation, sclerotium formation, ergosterol biosynthesis, and adaptation to hypoxic environments. These findings highlight the importance of lipid homeostasis and hypoxia adaptation in fungal pathogenesis and suggest that targeting *SRE1* could offer new strategies for managing grey mould disease in agricultural systems. For example, it may be possible to use *SRE1* (or its protein product) as a molecular target to inhibit conidiation and pathogenicity of *B. cinerea* through methods such as spray-induced gene silencing.

## 4. Materials and Methods

### 4.1. Fungal Strains and Culture Conditions

The strains used in this study are listed in Table S1. *B. cinerea* WT strain B05.10 and its derived strains, including knockout mutant ∆*sre1* and the complemented strain ∆*sre1*-c, were cultured on PDA or CM plates as previously described [33,34,35].

### 4.2. Gene Knockout and Genetic Complementation

Vectors and primers used in this study are listed in Table S2 and Table S3, respectively. The gene replacement method was used for *SRE1* knockout. The vector pXEH, containing the hygromycin resistance gene *HPH* [35], was used to construct the knockout vector. The 5′- and 3′- homologous flanks of *SRE1* were amplified and cloned into pXEH in the upstream and downstream of *HPH*, respectively. The resultant vector pSRE1-ko was transformed into *A. tumefaciens* strain AGL-1 as previously described [36]. The ATMT method was used to obtain fungal transformants as previously described with minor modification [37]. The cocultivation was performed on cellophane. The transformants were selected on PDA supplemented with 100 mg/L hygromycin.

The vector pXEG, resistant to G418, was used to generate the complemented strain ∆*sre1*-c. The full fragment of *SRE1* was amplified by PCR and cloned into pXEG to generate the complemented vector, which was then transformed into ∆*sre1* via the ATMT method. The resultant transformants were selected on PDA plates containing 50 mg/L G418 [38].

The transformants were screened by PCR amplification. The *SRE1* deletion mutants and complemented strains were further confirmed by qRT-PCR [2]. DNA and RNA were extracted as previously described [39,40].

### 4.3. Fungal Developmental Assays

The development analysis of *B. cinerea* strains were performed as previously described [36]. Briefly, the growth of the tested strains was determined by measuring the radial diameter of colonies in mm on PDA at 3 DPI. Conidial number per plate was calculated at 12 DPI of CM cultures. Conidial suspensions (10^5^ conidia/mL) in 1/2 potato dextrose broth (PDB) were used for the measurement of conidial morphology and germination. For sclerotial formation, strains were cultivated on CM at 20 °C in darkness for 15 DPI. For infection cushion observation, 10 μL conidial suspensions (10^5^ conidia/mL in 1/2 PDB) of the tested strains were inoculated on glass slides and cultured at 20 °C and observed at 16–24 DPI.

### 4.4. Pathogenicity and Penetration Assays

Pathogenicity and penetration assays were performed as previously described [34]. Briefly, mycelial plugs (5 mm in diameter) or conidial droplets (2 × 10^5^ conidia/mL in 1/2 PDB, 10 µL) were inoculated on green bean leaves, incubated in plastic containers with high humidity at 20–25 °C, and observed at 3 DPI. For the penetration assay, conidial droplets were inoculated on onion epidermis. After incubation, the inoculated epidermis was stained with lactophenol aniline blue and observed microscopically.

### 4.5. Hypoxia Adaptation Assays

Prepare conidial suspensions (2 × 10^5^ conidia/mL, containing 50 mM glucose) of the tested *B. cinerea* strains. The hypoxia-mimicking agent CoCl_2_ [31] was added at concentrations of 0, 100, 200 μM, respectively; incubate and observe conidial germinations at 4–8 HPI.

For analyzing the effects of hypoxia on mycelial growth, 2 mL conidial suspensions (1 × 10^6^ conidia/mL) of the tested *B. cinerea* strains were inoculated into 100 mL liquid CM supplemented with 0, 200 μM CoCl_2_ respectively, and incubated unshaking (with shaking as control of normoxia) for 7 days. Mycelia were harvested, dried at 45 °C for 24 h, and weighted for biomass quantification.

### 4.6. Quantification of Ergosterol

For ergosterol extraction, the tested strains of *B. cinerea* was cultured on PDA plates covered with sterile cellophane at 20 °C for 7 days. Mycelia were gently scraped off cellophanes, and total ergosterol was extracted and analyzed as previously described [41].

### 4.7. Imidazole Sensitivity Assay

Prepare conidial suspensions (2 × 10^5^ conidia/mL, containing 50 mM glucose) of the tested *B. cinerea* strains. Add 1 μL, 2 μL, 5 μL, or 10 μL of 1 mol/L imidazole solution to 1 mL conidial suspensions prepared above and mix well. Then, take 10 μL of each conidial suspension and place it on a glass slide and incubate them in a humid and dark condition at 20 °C. Conidial germinations were observed under a microscope at 4 HPI and 8 HPI.

### 4.8. Statistical Analysis

All the quantitative data presented in this study represent results from triplicate experiments independently performed at least three times. To easily compare the results from different independent experiments, the data of controls including mycelial growth, lesion size, conidiation, and so forth, were normalized as 1 or 100% in each independent experiment. The significance of the data was assessed using Student’s *t*-tests. And the *p*-value lower than 0.05 was considered to be statistically significant.

## Data Availability

All data included in this study are contained within the article and Supplementary Materials [35,38,42].

## Supplementary Materials

The following supporting information can be downloaded at: https://www.mdpi.com/article/10.3390/ijms26031365/s1.
