# Supplementary material for: Sterol Regulatory Element-Binding Protein Sre1 Mediates the Development and Pathogenicity of the Grey Mould Fungus Botrytis cinerea
Source: Int J Mol Sci. 2025 Feb 6;26(3):1365. doi: 10.3390/ijms26031365 (PMC11818819; doi:10.3390/ijms26031365)
Supplement: Supplementary file 1 [file ijms-26-01365-s001.zip › ijms-3364664-supplementary.pdf]

Supplementary Materials

Article title: Sterol regulatory element-binding protein Sre1 mediates development and pathogenicity of the grey mould fungus *Botrytis cinerea*

Authors: Ye Yuan, Shengnan Cao, Jiao Sun, Jie Hou, Mingzhe Zhang, Qingming Qin and Guihua Li

The following Supplementary Materials is available for this article:

Figure S1. Sensitivity of *Botrytis cinerea* strains to antifungal drug imidazole.

Table S1. Strains of *B. cinerea* used in this study.

Table S2. Plasmid vectors used in this study.

Table S3. Primers used in this study.

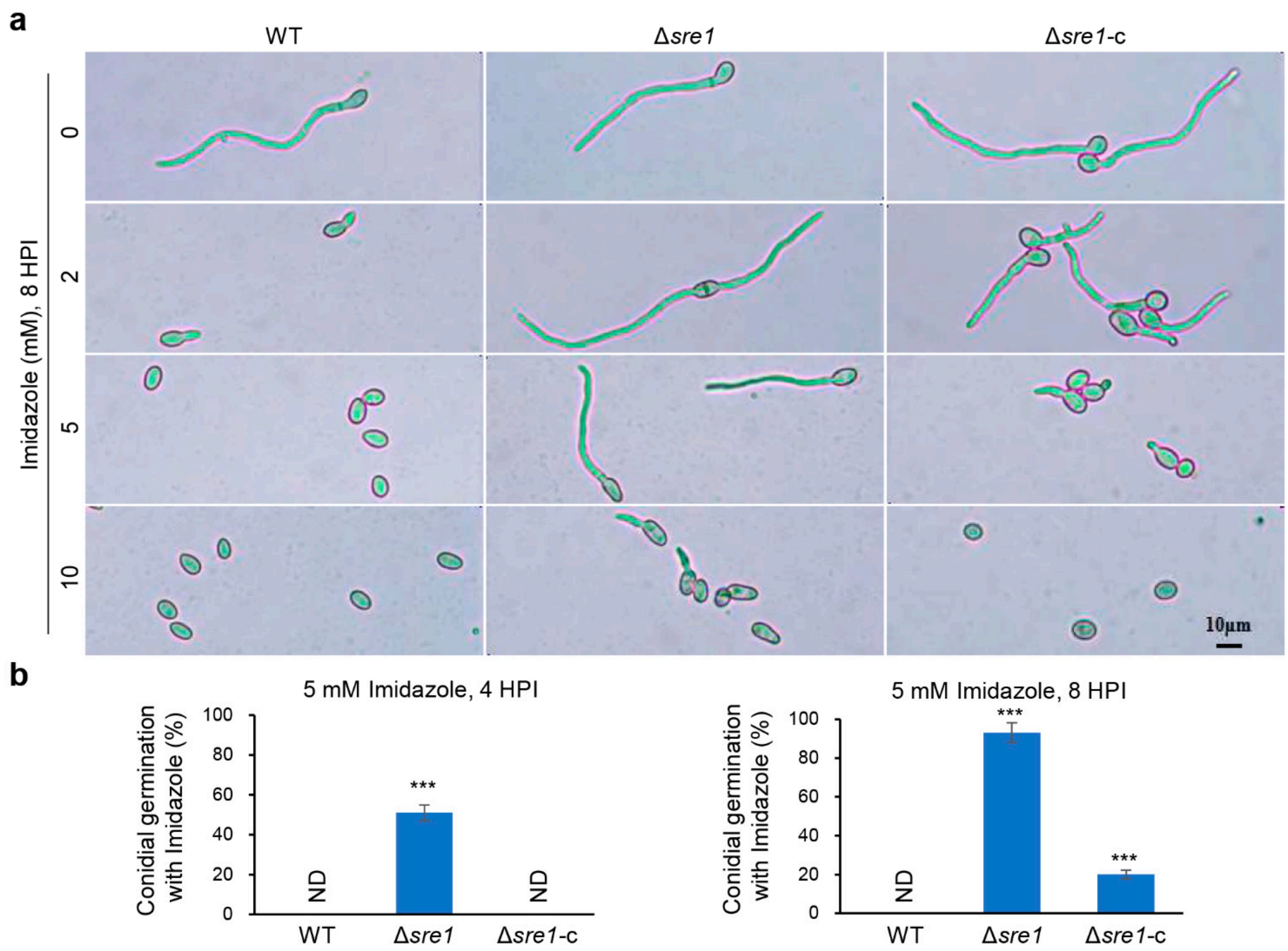

Figure S1. Sensitivity of *B. cinerea* strains to antifungal drug imidazole. Conidial suspensions (containing 50 mM glucose) of each strain were treated with 0, 2, 5, and 10 mM imidazole, respectively. (**a**) Conidial germinations were observed at 8 hours post inoculation (HPI). (**b**) Quantification of conidial germination treated with 5 mM imidazole at 4 HPI or 8 HPI. ND, Not detected.

Table S1 Strains of *B. cinerea* used in this study

Strain	Description	Source
B05.10	Wild-type strain	Quidde et al., 1998
Δ*sre1*	B05.10 with *SRE1* knocked out, Δ*sre1*::*HPH*	This study
Δ*sre1*-c	The ectopic complementary strain of Δ*sre1*, G418^R	This study

Table S2 Plasmid vectors used in this study

Vector	Description	Source
pXEH	Binary vector used for knockout of fungal genes, containing *HPH* gene within its T-DNA region; Km^R	Feng et al., 2017
pXEG	Binary vector used for knockout of fungal genes, containing *NPTII* within its T-DNA region; Km^R	Tang et al., 2024
pSRE1-ko	Constructed from pXEH, for knockout of *SRE1*	This study
pSRE1-c	Constructed from pXEG, for genetic complementation of Δ*sre1*	This study

Table S3 Primers used in this study

Primer Name	Primer sequence (5’- 3’)	Application
SRE1-UF	GAATTCCGCCTCCCTTGGTAGATAG	Construction of *SRE1* knockout vector
SRE1-UR	GGTACCGTCGTGGGTGGTCTGATTT	
SRE1-DF	GGATCCGGATACCAGGTAAGCGAATG	
SRE1-DR	AAGCTTCTCACCAACCAACCAAAACA	
RE-F	GGAAGGCTTTTCGAGGTCA	Screening *SRE1* knockout strains
RE-R	GACGAAAAGACGCCGAAGA	
Ha-RC	ATGATGCAGCTTGGGCGCA	
Hb-RC	ACAGACGTCGCGGTGAGTTCA	
RI-F	AACCTTTGCTGTTCCCACG	
RI-R	CAACATCGCCACCAGTCAG	
SRE1-CF	GATTCGTGTCATTAGGGTTCATTCTT	Complementation of Δ*sre1*
SRE1-CR	GATTCCTGTCGGTAACTTTGATTGGT	
ACT-F	CATGGCTGGTCGTGATTTGA	qRT-PCR for Actin gene
ACT-R	GAGGATTGACTGGCGGTTTG	
